# New epidemic cluster of pre-extensively drug resistant isolates of *Mycobacterium tuberculosis* Ural family emerging in Eastern Europe

**DOI:** 10.1186/s12864-018-5162-3

**Published:** 2018-10-22

**Authors:** Viacheslav Sinkov, Oleg Ogarkov, Igor Mokrousov, Yuri Bukin, Svetlana Zhdanova, Scott K. Heysell

**Affiliations:** 1 Department Epidemiology and Microbiology, Scientific Centre of the Family Health and Human Reproduction Problems, 16 Timiriazeva street, Irkutsk, 664003 Russia; 2 Irkutsk State Medical Academy of Continuing Education, Irkutsk, 664049 Russia; 3 Laboratory of Molecular Epidemiology and Evolutionary Genetics, St. Petersburg Pasteur Institute, 14 Mira street, St. Petersburg, 197101 Russia; 4 Limnological Institute SB RAS, Irkutsk, 664033 Russia; 5 Irkutsk National Research Technical University, Irkutsk, 664074 Russia; 6 Division of Infectious Diseases and International Health, University of Virginia, Charlottesville, VA 22908 USA

**Keywords:** *Mycobacterium tuberculosis*, Ural genotype, Molecular epidemiology, Molecular evolution, Phylogeography, Phylogenetics, Next generation sequencing, Population genetics

## Abstract

**Background:**

Ural genetic family is a part of the Euro-American lineage of *Mycobacterium tuberculosis* and is endemic in Northern Eurasia (former Soviet Union [FSU]). These strains were long described as drug susceptible and of low virulence, but recent studies reported an increasing circulation of the multidrug-resistant (MDR) and extensively drug-resistant (XDR) Ural strains. Here, we analyzed all publicly available whole genome sequence data of Ural genotype isolates, in order to elucidate their phylogenomic diversity with a special focus on MDR and potentially epidemic clones.

**Results:**

A total of 149 *M. tuberculosis* genomes of Ural isolates from FSU countries were mined from the GMTV database and TB-ARC project. We identified 6002 variable amino acid positions that were assessed for functional significance and used to build ML, NJ trees and for Bayesian TMRCA estimation. Three robust monophyletic clades were identified: Clade A (31 isolates from Russia, Belarus, Moldova), Clade B (52 isolates from Russia), and Clade C (37 isolates from Moldova, 2 from Belarus). Clade C was significantly associated with XDR or pre-XDR status compared to the pooled Clades A and B (33/39 versus 5/83, *P* < 0.0001). Time of origin was estimated for Clade A at 77.7–137 years ago and for Clade B at 56.3–99.2 years ago compared to the significantly more recent origin for Clade C. in silico spoligotyping identified signatures specific of the Clade A (spoligotype SIT35), and Clades B and C (both SIT262).

**Conclusions:**

A genetically compact and evolutionarily young Ural Clade C, likely originated after collapse of the Soviet Union, and reached epidemic proportions in Moldova in the last 20 years. This epidemic pre-XDR clone (mostly rifampin, isoniazid and kanamycin resistant) is characterized by a specific combination of mutations: KatG Ser315Thr, *fabG1* -15C > T, RpoB Ser450Leu, RpsL Lys88Arg, *eis* -12G > A and EmbB Ser297Ala/T > G. Its further dissemination may occur towards both Russia and European Union and should be taken into consideration by health authorities. The identified spoligotyping signatures can serve for rapid preliminary detection and surveillance of the more hazardous pre-XDR associated strains of the Ural family, both in populations from countries of their endemic circulation and migrant communities.

**Electronic supplementary material:**

The online version of this article (10.1186/s12864-018-5162-3) contains supplementary material, which is available to authorized users.

## Background

*Mycobacterium tuberculosis* is a clonal bacterial species with hierarchical population structure made up of 8 phylogenetic lineages (as per current knowledge). The Ural genetic family is a part of the large and heterogeneous Euro-American lineage (lineage 4). The Ural family was initially proposed by variable number tandem repeat (VNTR) analysis [1]. More recently, its characteristic spoligotyping signature [2] and specific single nucleotide polymorphism (SNP) in the *Rv1811* gene [3] were described.

Phylogeographically, the Ural family is mainly circumscribed to Northern Eurasia, i.e. Russian Federation and countries of the former Soviet Union (FSU), with sporadic isolates found elsewhere [4, 5]. In Russia, the prevalence of the Ural genotype was reported at 5–12% [4] and it has long been of less public health concern compared to regionally common Beijing and LAM families [2, 4]. Compared to the Beijing genotype, Ural strains were shown less virulent both in experimental models [6], and based on study of autopsy samples of patients that died from tuberculosis (TB) [7]. Regarding drug resistance, meta-analysis of studies from former Soviet Union demonstrated a significantly decreased odds of being multidrug-resistant (MDR) for Ural compared to the Beijing strains (*P* < 0.00001; pooled OR 0.22; 0.11–0.41) [5]. However, more recent studies from different parts of Eastern Europe (Moldova, Lithuania, northwestern Russia) reported an increasing circulation of MDR Ural strains with concern for further amplified resistance, such as pre-extensively or extensively drug-resistant (pre-XDR, XDR) ([5, 8, 9], and references therein).

Due to its limited circulation beyond Russia and the previously believed “less-hazardous” pathogenic profile, no large-scale genomic studies have specifically focused on these strains to date. Here, we analyzed the publicly available whole genome sequencing (WGS)/next generation sequencing (NGS) data of the Ural genotype isolates, in order to elucidate the phylogenetic diversity of this family with special focus on the potentially epidemic and MDR clonal groups. In addition, we sought to identify a robust spoligotyping signature of the main phylogenetic clades within the Ural family.

## Results

### WGS insight into Ural family: Clustering and drug resistance

**Fig. 1:**
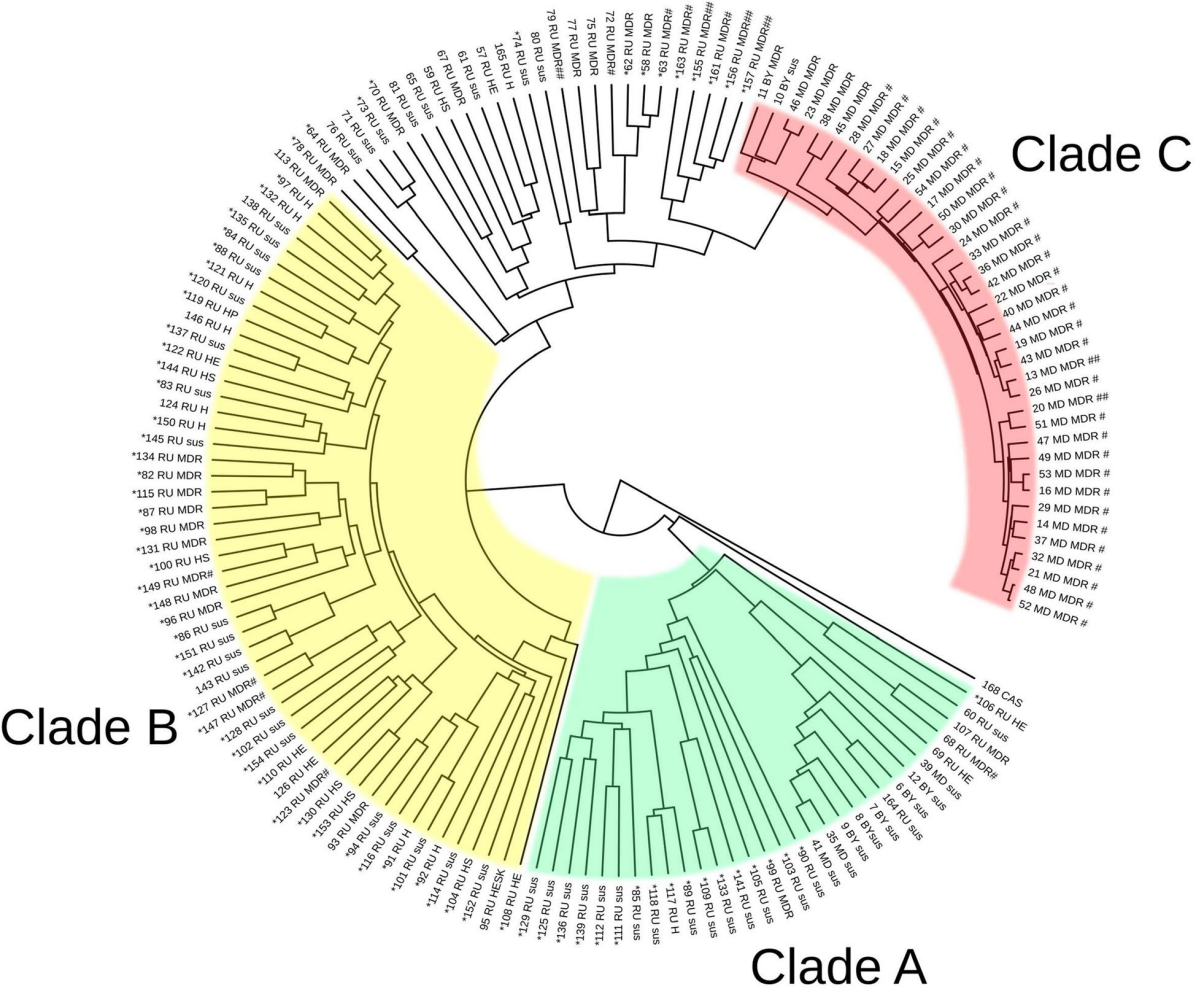
ML consensus phylogenetic tree of 149 M. tuberculosis Ural family strains (CAS family strain was used as outgroup) based on 6002 genome-wide variable amino acid positions. Strain designation includes consecutive number (Additional file 1: Table S1), country of origin, drug resistance. Abbreviations: RU – Russia, MD – Moldova, BY – Belarus, MDR – multidrug resistance, H – isoniazid resistant, E – ethambutol resistant, S – streptomycin resistant, P - pyrazinamide resistant, sus – susceptible, # - pre-XDR, ## - XDR. *- genotypic resistance profile confirmed by phenotypic data (when available at GMTV database http://mtb.dobzhanskycenter.org/). The CAS strain was used as outgroup under phylogenetic analysis; it did not have any drug resistance mutation

The study collection included WGS/NGS data of 149 *M. tuberculosis* Ural family isolates from Russia, Belarus and Moldova whose analysis identified 6002 variable amino acid positions that were used to build the phylogenetic ML-tree (Fig. 1, Additional file 1: Table S1, Additional file 2, Additional file 3). The shaded parts on the dendrogram depict three robust clades: Clade A (23 isolates from Russia, 3 from Moldova, 5 from Belarus), Clade B (52 isolates from Russia), and Clade C (37 isolates from Moldova and 2 from Belarus) (Fig. 1). Clades were defined based on the most basal branch with double support by bootstrap test with 100 iterations (0–100) and fast likelihood-based distance method in 1000 iterations (0 to 1) (Additional file 2: Figure S1). Accordingly, the strains without double support were defined as ‘out-of-clade’ strains and were excluded from further analysis. It may be noted that 57% of these out-of-clade strains were MDR (Table 1, Additional file 1: Table S1) and are likely related to Clades B or C. Comparison of the three clades with regard to the MDR properties revealed an overwhelming MDR prevalence at 94.9% in the Clade C and the lowest MDR prevalence in the Clade A (Table 1, Additional file 1: Table S1). An intermediate MDR prevalence was found in the Clade B and out-of-clade strains.

**Table 1:** Distribution of drug resistance among phylogenetic clades of the Ural family

Identifiers	Number of strains (*n* = 149)	Susceptible and monoresistant, number (%)	Polyresistant, number (%)	MDR, number (%)	Pre-XDR and XDR, number (%)	Pre-XDR and XDR prevalence in Clades C vs A&B, χ2^a^; p
Out-of-clade strains	27	9 (33.3)	2 (7.4)	16 (59.3)	8 (29.6)	65.3; < 0.0001
Clade A	31	26 (83.9)	2 (6.4)	3 (9.7)	1 (3.2)	
Clade B	52	28 (53.9)	10 (19.2)	14 (26.9)	4 (7.7)	
Clade C	39	1 (2.6)	0	38 (97.4)	32 (82.1)	

^a^**χ2** with Yates correction

Additional analysis of the mutations associated with resistance to the second-line drugs permitted us to genotypically assess MDR strains as having further potential resistance: pre-XDR or XDR (Additional file 1: Table S2). In total, 7 isolates were XDR and 40 were pre-XDR. Of those latter, 38 were pre-XDR due to additional resistance to the injectable aminoglycoside (mostly kanamycin) and 2 were pre-XDR due to resistance to fluoroquinolones. The data on phenotypic 2nd line drugs resistance profile were scarce for the studied strains and were available only for some isolates in the GMTV database. For this reason, we did not compare phenotypic versus genotypic resistance to 2nd line drugs for XDR and pre-XDR strains.

XDR strains can be found in different parts of the dendrogram and were not associated with any clade (Fig. 1). In contrast, Clade C consisted predominantly of pre-XDR isolates (33/39) and all of them were from Moldova. Comparison of the three clades revealed a significant association with XDR or pre-XDR status of the Clade C compared to pooled Clades A and B (33/39 versus 5/83, *P* < 0.0001; OR 84.7 95% CI: 24.1 to 297.1).

A closer look into molecular basis of drug resistance (Table 2, Additional file 4) revealed that *katG315,* associated with high level isoniazid resistance and *rpoB450* (*rpoB531* according to *E. coli* numbering system), associated with rifampin resistance, were the most frequent in all clades. As for streptomycin resistance, the most frequently globally mutated *rpsL43* was however found in minority of isolates. In contrast, there was an increasing trend of prevalence of the *rpsL88* mutation from Clade A to Clade C.

**Table 2:** Prevalence of mutations in different clades of the Ural genotype

Gene, codon or position	Clade A (*n* = 31)	Clade B (*n* = 52)	Clade C sensu stricto (*n* = 33)	Clade C^a^ (*n* = 39)	Other (*n* = 27)
rpoB					
Ser450Leu	2	12	31	34	15
445	1	1			1
452		1			
435			1	1	
Ser450Trp			1	1	
rpoC Asp485Asn		7			4
katG Ser315Thr	5	33	33	38	20
fibG1 (inhA) -15 C > T		7	33	34	5
ahpC promoter	1	1			
rpsL					
43		6		1	1
88	2	10	33	35	11
embB					
297			29	29	2
306	1	6	1	2	7
319		2			
406		2	1	3	1
497		3	2	2	3
1002		1			
1024				2	
embA					
-12 C > T	1				
-16 C > T			8	8	
rrs					
517		1			
1401					2
eis					
-12G > A	1	3	33	33	3
-15G > C		2			1
-37C > A		1			
-10C > T					1
gyrA					
90			1	1	2
94		1	1	1	4
pncA					
13			1	1	
160			1	1	
-7			1	1	
12			4	4	
14			2	2	
-11			1	1	2
34			1	1	
128		1			1
10				2	
159					1

^a^Clade C includes “Clade C strict sense” plus pre-Clade C isolates (refer to different rose shade on dendrogram in Additional file 5). In few strains, two *embB* mutations were found

On a whole, Clade C, consisting mainly of isolates from Moldova, was marked by the peculiar combination of drug resistance mutations. While some of them are known to be globally prevalent overall (*rpoB450*, *katG315*, *inhA* − 15), some other mutations were less frequently (*rpsL88*, a gene associated with streptomycin resistance and *eis* -12G > A, a promotor region associated with kanamycin resistance) or rarely (EmbB Ser297Ala, *embA* -16C > T, genes associated with ethambutol resistance) described. On the example of the very homogeneous and apparently recently emerged group that we have thus named as ‘Clade C sensu stricto’ (only isolates from Moldova) it is possible to observe a stepwise pattern of acquisition of mutations (Additional file 4). Thus all 33 these isolates have the following mutations: KatG S315 T/C > G, *fabG1* -15C > T, RpoB Ser450Leu/C > T, RpsL Lys88Ar/ A > G, *eis* -12G > A. In addition, 29 of 33 have further acquired EmbB Ser297Ala/T > G, and 8 more isolates - *embA* -16C > T (8 isolates). Finally, 7 different mutations in *pncA* (the gene associated with the majority of phenotypic resistance to pyrazinamide) were found in 11 isolates. Interestingly, *eis* -12G > A mutation was found not only in all 33 ‘Clade C sensu stricto’ isolates but also in 7 more isolates found across other parts of the tree. On the other hand, EmbB Ser297Ala/T > G and *embA* -16C > T were found exclusively in this branch.

After these findings related to the identified Ural clades, we sought to additionally examine other available Ural genomes from Iran and Romania. We found that two Iranian Ural strains had identical resistance mutations EmbB Ser366Pro, GyrA Asp94Gly, and RpoB Gln436Leu. On the other hand, 4 of 5 Romanian Ural isolates were genotypically drug susceptible while 1 isolate was isoniazid-resistant due to katG Asp311Glu mutation. Thus, Ural isolates from Iran and Romania did not bear any resistance mutation found among Ural strains in Clade C. A cluster analysis based on vcf files provided by the Broad institute revealed that strains from Iran and Romania clustered into two separate branches and both were beyond isolates from FSU (not shown).

### WGS insight into Ural family: Phylogenomics

Testing different models of amino acid substitutions revealed the JTT + G model with gamma correction g = 0.802 to be the best one. The BIC value for this model was 317,852.64. In the list of models available in the BEAST v.1.8.2 program, the JTT + G model with BIC 322153.12 was the closest to the best fit model and was thus retained for further NJ and Bayesian analysis.

**Fig. 2:**
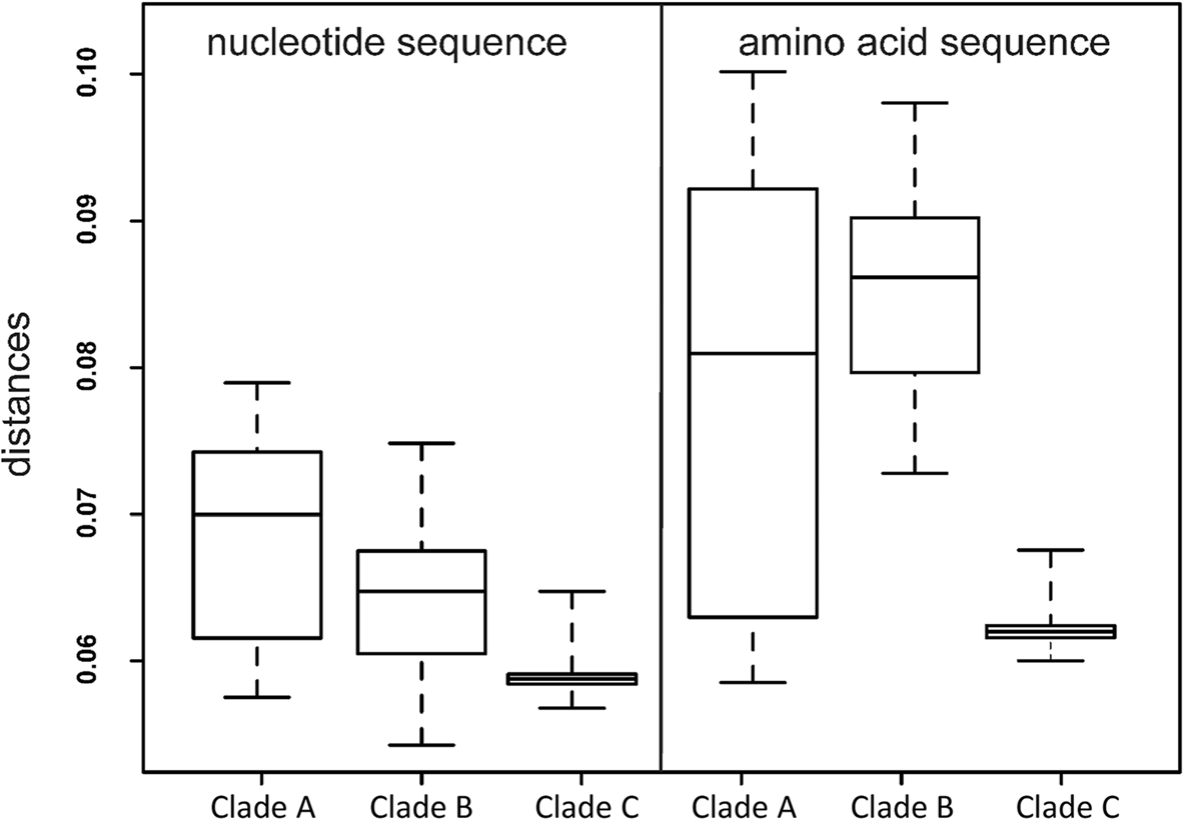
Distribution of distances between amino acid and nucleotide sequences in three Ural clades

The interclade distances (Fig. 2) were compared for significance using Wilcoxon-Mann-Whitney test (Table 3). This analysis demonstrated a significant difference between Clades C vs B and C vs A. Thus nonsynonymous nucleotide amino acid sequences in different clades had acquired a different number of substitutions since the most recent common ancestor. This suggests that strict molecular clocks are not suitable for phylogenetic reconstruction due to different mutation rate in the identified Ural clades.

**Table 3:** Pairwise clade comparison by Wilcoxon-Mann-Whitney test

	Clade C	Clade B	Clade A
Clade C	–	*P* < 0.01	P < 0.01
Clade B	W = 0	–	*P* < 0.05
Clade A	W = 209	W = 987	–

Upper right triangle – probability of rejecting null hypothesis about lack of significant difference between mean mutation rates (amino acid substitutions) in three Clades. Lower left triangle shows Wilcoxon-Mann-Whitney statistics values. Lognormal relaxed clock model was used for calculations

Accordingly, we used a Lognormal relaxed clock model for Bayesian phylogenetic analysis with BEAST. The effective sample size (ESS Statistics) was determined to be from 421.5 to 1367.9, i.e. exceeded the significance threshold of 200. All three clades with respective strain content remained unchanged in the Bayesian phylogenetic tree correlating with ML and NJ trees (Fig. 1, Additional file 2). This reconfirms the robustness of the above analysis and monophyletic nature of these clades.

Mean mutation rate in *M. tuberculosis* was previously estimated to range from 0.21 × 10^− 8^ to 5.97 × 10^− 8^ per nucleotide per year [10]. We used this estimation to assess time of the clade divergence with 95% CI that resulted in TMRCA from ten to 300 years. To reduce uncertainty, we assumed that the most recent Clade C dominated by strains from Moldova, originated 25 years ago, coinciding with the collapse of the Soviet Union and establishment of the independent Republic of Moldova. Based on this assumption, time of origin was estimated for Clade B 56.3–99.2 years ago, and for Clade A 77.7–137 years ago.

### Spoligotyping signatures of the Ural clades

We performed in silico spoligotyping of the NGS data and compared the obtained profiles to the WGS dendrogram (Additional file 5). Low sequencing depth of the CRISPR locus prevented reliable reconstruction of spoligotyping profiles of some isolates. Nonetheless, it was possible to identify specific signatures (i.e. deleted signals) for particular clades. In particular, mainly drug susceptible Clade A correlates with SIT35 and derived profiles, while mainly or overwhelmingly MDR Clades B and C correlate with SIT262 and derived profiles. The Ural founding spoligotype SIT777 was identified in a single strain (Additional file 5); it is equally distant from all clades which underlines its ancestral position as Ural prototype spoligoprofile. When calculated for isolates with available spoligotypes, the rate of MDR strains in SIT262 (and derived profiles) was significantly higher than in SIT35 (and derived profiles): 50/96 vs 5/24 (*P* = 0.009; OR 4.13 [95% CI: 1.42–1.96]).

## Discussion

The present study focused on the underestimated genetic family of *M. tuberculosis*, the Ural genotype, which has been increasing in circulation in some parts of Eastern Europe, including northwestern Russia, and has furthermore been accompanied by amplifying drug resistance. The availability of the large NGS dataset permitted us to gain insight into both distant evolutionary history and current epidemiology of this family.

The reconstruction of evolutionary history of the phylogenetic lineages and clones including dating their origin and hallmark events is not a straight and smooth highway. This analysis should combine rigorous computation and common sense and take into consideration the bottleneck events that could have led to extinction of the first parental strain. Consequently, the estimated timing is based on coalescence analysis of extant lineages and thus presents TMRCA of these extant lineages [11]. In this view, seemingly contrasting results are not contradictory but just address different questions: origin of the first strain in the lineage (that may be extinct) versus origin of the currently predominant circulating strains. For example, RD-Rio sublineage within the Latin American Mediterranean family could have originated in West Africa but its epidemic circulation and primary dispersal started in Brazil [12]. Thus the Ural family was estimated to originate in the area north of the Black Sea > 1800 years ago [4, 5, 13] and this dating seems at odds with our estimation of 150–300 years ago. However only a single strain with prototype Ural spoligotype SIT777 was available in the studied dataset and consequently the estimated time is TMRCA of the currently circulating and derived clades, Clade A (SIT35) and Clades B and C (SIT262). An active circulation of the older Clades A and B may have started at the end of the nineteenth century and for some reason both may have become predominant compared to the prototype and other hypothetical sister clades within Ural. Similar is the situation with the well-known Beijing genotype that includes ancient strains (genetically more diverse, less numerous) compared to globally widespread modern Beijing sublineages. In its turn, some more epidemiologically or clinically relevant clusters have emerged within the modern Beijing strains and their clinical relevance have been demonstrated in spinal TB [9] or TB-HIV co-infected patients [14, 15].

Regardless of the definitive TMRCA estimation, in relative terms Clade C is the least diverse and the evolutionarily youngest group (Fig. 1, Fig. 2). On the other hand, two “older” Clades A and B have overlapping confidence intervals (Fig. 2) and it was not possible to reliably decide which of them originated earlier. We hypothesize that both Clades A and B started their circulation within the same time period, likely at the end of the 19th – beginning of the twentieth century, coinciding with or fueled by accelerated industrialization in the Russian Empire. Based on comparison with in silico spoligotyping profiles, it also appears that Clades A and B emerged at approximately the same time within nearly extinct parental population and followed different courses of evolution with regard to acquisition of MDR. On the other hand, the most recent clade C originated within the larger population of the Clade B (as both of them share a SIT262 signature).

It should be noted that all resistance mutations were identified as such according to the TB Profiler database [16] and also described in other resources. However, the prevalence of such mutations and their capacity to confer high-level resistance is different. In the most recent large study of > 6000 isolates [16], both promoter mutations *eis* -12G > A and *embA* -16C > T were defined as high frequency (> 10 isolates) while EmbB Ser297Ala was not described at all. In the large earlier study, *embA* -16C > T mutation was described as conferring ethambutol resistance at 10–40 μg/ml and found in 2/75 ethambutol-resistant isolates, but it co-occurred with other *embCAB* mutations [17]. In the same study, *embB297* mutation was described in 1/75 ethambutol-resistant strains and it was not accompanied by other *embCAB* mutations. Both of these mutations were in strains from Russian migrants in USA. EmbB Ser297Ala is included in PHYRESSE online tool but is not labeled as high-confidence SNP for ethambutol resistance.

Beyond pre-XDR mutations found in Clade C, the additional finding of mutations in *rpoC* is also noteworthy. Mutations in *rpoA* and *rpoC*, while not associated with rifampin resistance, are rather known to confer compensatory mechanisms for the otherwise reduced fitness cost of major *rpoB* mutations [18]. One such mutation RpoC Asp485Asn was found in 11 isolates in the studied collection. While 7 of these isolates made a compact cluster within the intermediate Clade B, 4 other were different and found in distant out-of-clade isolates. All 11 isolates with RpoC Asp485Asn were MDR, of them 1 was pre-XDR, and 1 was XDR.

An independent occurrence of putative resistance mutations (*eis* -12G > A, RpoC Asp485Asn) in different phylogenetic branches suggests their true causative link with resistance/adaptation. Relatedly, *eis* -12 T mutant allele was shown in a transduction model to confer resistance to kanamycin (but not to amikacin or capreomycin ([19], and Suporn Pholwat, personal communication). However, a geographic variation of such mutations also influenced by the genetic background of the strain, may also occur. For example, *rpoC485* mutations were not described in the Chinese study dominated by the Beijing genotype strains [20]. On the other hand, this RpoC Asp485Asn was described by Comas et al. [18] in Beijing isolates in high MDR-TB burden areas in the Former Soviet Union (Georgia, Kazakhstan, Uzbekistan), and was defined as a “high-probability compensatory mutation.”

## Conclusions

A genetically compact clone of the *M. tuberculosis* Ural family marked with a particular combination of drug resistance mutations (KatG Ser315Thr, *fabG1* -15C > T, RpoB Ser450Leu, RpsL Lys88Arg, EmbB Ser297Ala/T > G and *eis* -12G > A) emerged and reached pre-XDR status and epidemic proportions in Moldova in the last 20–25 years. The absence of this Clade C in Russia (at least in the studied NGS dataset) may be explained by its recent emergence. The wider dissemination of this potentially epidemic pre-XDR clone beyond Moldova may take place towards both Russia and European Union thus leading to increase of primary MDR-TB (in patients without a prior history of anti-TB treatment) and should be taken into consideration by their health authorities.

However, the combination of mutations specific of the Ural ‘Clade C sensu stricto’ (KatG S315 T/C > G, *fabG1* -15C > T, RpoB Ser450Leu/C > T, RpsL Lys88Ar/ A > G, *eis* -12G > A) cannot serve alone for reliable identification of this pre-XDR group. EmbB Ser297Ala was identified in two Ural strains from Russia in this study while *embA* -16C > T was detected in four Beijing genotype isolates in GMTV database. They are not neutral mutations. Overall, some of them are well-known and frequent (*rpoB450* = *rpoB531*, *katG31*, *inhA* − 15), some other are less frequent (*eis* − 12, *embB297*) and it is possible that such mutations may be selected in other Ural isolates beyond Clade C and in other phylogenetic lineages of *M. tuberculosis*. Therefore, we would rather caution against using resistance mutations as markers of phylogenetic clades. Speculatively, this combination of mutations may be regarded in the context of their hypothetical epistatic interaction and future studies are warranted.

The WGS implemented within the personalized medicine approach represents the current state of practice for many developed countries and an aspirational technology for other settings. As such, WGS is not likely to be totally implemented at the global scale or for individualized patient care in the near future. Therefore, availability of a molecular signature for highly drug-resistant and epidemiologically relevant *M. tuberculosis* clones using non-expensive and simpler methodologies remains desirable. In this view, SIT35 and SIT262 may serve as useful proxies for rapid preliminary detection and surveillance of the less and more hazardous branches, respectively, of the Ural family, both in the countries of their endemic circulation and in migrant communities.

## Methods

### *M. tuberculosis* strains/genomes

The genomes of the *M. tuberculosis* Ural family strains were searched in the GMTV database [21] and in genomes downloaded from TB-ARC project (https://olive.broadinstitute.org/projects/tb_arc). Specific mutations in *Rv1811* and *Rv0684* previously described by Homolka et al. [3] and Coll et al. [22], respectively, served as Ural family SNP markers. The whole genome short sequencing reads were downloaded from Sequence Read Archive (NCBI NIH, USA) as sra files and converted to fastq format by fastq-dump program (ver. 2.4.0) (SRA Toolkit, NCBI NIH, USA).

As a result, 156 Ural family genomes were identified. Further analysis was performed on 149 Ural family isolates from FSU countries (Additional file 1: Table S1) while Ural family samples from Romania (*n* = 5) and Iran (*n* = 2) were not included due to small sample size.

### Bioinformatics analysis

Short reads were mapped to the reference genome of *M. tuberculosis* H37Rv (NC_000962) [23] by using the Burrows-Wheeler Aligner v. 0.7.12 [24]. Single nucleotide polymorphism (SNP) calling was performed by SAMtools v.0.1.19 [25]. Variable call format (vcf) files were annotated by snpMiner2 v.2 [26]. Multiple alignment sequences were made by Mafft v.7 [27]. Drug resistance mutations were taken from Tuberculosis Drug Resistance Mutation Database [28] and from TB Profiler database (http://tbdr.lshtm.ac.uk/). MDR, pre-XDR and XDR phenotypes were defined according to the World Health Organization definitions [29]: MDR are strains resistant to isoniazid and rifampicin; XDR - resistant to isoniazid, rifampicin, fluoroquinolone, and a second-line injectable agent; pre-XDR - resistant to isoniazid, rifampicin and either a fluoroquinolone or a second-line injectable agent.

For GMTV strains, genotypically deduced drug resistance was compared with phenotypic profile when available (Fig. 1, Additional file 1: Table S1).

Information on the functional category of genes was taken from TubercuList (https://mycobrowser.epfl.ch/) and *M. tuberculosis* H37Rv gene list [30].

ProTest v. 3.2 program [31] served to select model of acquisition of amino acid substitutions in order to calculate genetic distances. Preliminary phylogenetic reconstruction and delineation of stable clades were performed by PhyML 3.0 [32] based on maximum likelihood (ML) algorithm. Mann-Whitney-Wilcoxon criterion [33] was used to test for significant difference between clades. NJ algorithm with bootstrap analysis [34] additionally served to assess robustness of the phylogenetic reconstruction and topology of the dendrogram. Bayesian phylogenetic analysis with molecular clock was done with BEAST v.1.8.2 [35]. *jModelTest* 2.2.7 program [36] was used to select the model of nucleotide substitutions using Bayesian information criterion (BIC). The amino acid sequence of the total proteome of the CAS (Central-Asian family) strain with accession number #ERR233347 was used as outgroup for building the phylogenetic tree as the most distant isolate with regard to the Ural family. The strict or relaxed molecular clock models were selected based on testing significant differences between amino acid based distances between outgroup and Ural clades. Mann-Whitney-Wilcoxon criterion [33] was used to test for significant difference.

Lognormal relaxed clock was used in the BEAST analysis. The MCMC results were examined with Tracer 1.8.2. The consensus tree was built with Tree Annotator while the first 15.3% of saved trees were discarded as burn-in.

The R package [37] was used for statistical analysis and visualization of distribution of distances. MEGA version 6.0 program [38] was used for previous phylogenetic analysis of the polymorphic loci and groups p-distances. Graphical presentation of trees was done using TreeGraph [39] and iTOL v3 [40].

The in silico spoligotyping was performed on short sequencing reads using SpoTyping program (https://github.com/xiaeryu/SpoTyping) [41].

## Additional files

Additional file 1: **Table S1.** Information on 149 *M. tuberculosis* Ural strains included in this study. Strain ID, drug susceptibility, country of isolation, Ural clades, Accession number, MDR associated mutations in *katG*, *rpoB*, *inhA*, *embB*. **Table S2.** Genotypic drug resistance profiles and mutations in the studied Ural strains. Strains are grouped in the same order as in Additional file 4: Figure S3 and Additional file 5: Figure S4, and the same color code for clades apply. (XLSX 26 kb)

Additional file 2: **Figure S1.** ML consensus phylogenetic tree of 149 *M. tuberculosis* Ural family and one CAS genomes based on genome-wide 6002 variable amino acid positons and tested for robustness by bootstrap test in 100 iterations (from 0 to 100) and fast likelihood-based distance method in 1000 iterations (from 0 to 1). Strain designation includes consecutive strain number (Additional file 1: Table S1), country of origin, drug resistance. (PDF 954 kb)

Additional file 3: **Figure S2.** Venn diagram of clade-unique and clade-shared amino acid substitutions. (PPTX 146 kb)

Additional file 4: **Figure S3.** WGS-based dendrogram of the Ural strains with added resistance profiles and mutations (see also Additional file 1: Table S2). (PPTX 2084 kb)

Additional file 5: **Figure S4.** WGS-based dendrogram of the Ural strains with added spoligotyping profiles. SIT – spoligotype international type according to SITVIT_WEB. Spoligotype signatures specific of the entire Ural family and its specific clades are shown in different colors. (PPTX 2071 kb)

## Abbreviations

BIC: Bayesian information criterion
FSU: Former Soviet Union
MDR: Multidrug-resistant
ML: Maximum likelihood
NGS: Next generation sequencing
SNP: Single nucleotide polymorphism
TB: Tuberculosis
TMRCA: Time to most recent common ancestor
VNTR: Variable number of tandem repeats
WGS: Whole genome sequencing
XDR: Extensively drug-resistant
