# Supplementary figures and images for: New epidemic cluster of pre-extensively drug resistant isolates of Mycobacterium tuberculosis Ural family emerging in Eastern Europe
Source: BMC Genomics. 2018 Oct 22;19:762. doi: 10.1186/s12864-018-5162-3 (PMC6198502; doi:10.1186/s12864-018-5162-3)

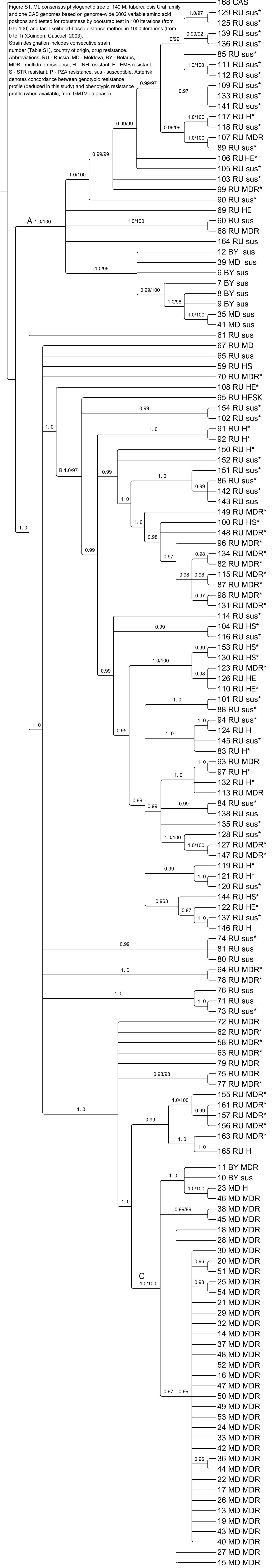

Supplement: Supplementary file 2 — Figure S1. ML consensus phylogenetic tree of 149 M. tuberculosis Ural family and one CAS genomes based on genome-wide 6002 variable amino acid positons and tested for robustness by bootstrap test in 100 iterations (from 0 to 100) and fast likelihood-based distance method in 1000 iterations (from 0 to 1). Strain designation includes consecutive strain number (Additional file 1: Table S1), country of origin, drug resistance. (PDF 954 kb) [file 12864_2018_5162_MOESM2_ESM.pdf]
