# Supplementary material for: New epidemic cluster of pre-extensively drug resistant isolates of Mycobacterium tuberculosis Ural family emerging in Eastern Europe
Source: BMC Genomics. 2018 Oct 22;19:762. doi: 10.1186/s12864-018-5162-3 (PMC6198502; doi:10.1186/s12864-018-5162-3)
Supplement: Supplementary file 3 — Figure S2. Venn diagram of clade-unique and clade-shared amino acid substitutions. (PPTX 146 kb) [file 12864_2018_5162_MOESM3_ESM.pptx]

## Slide 1
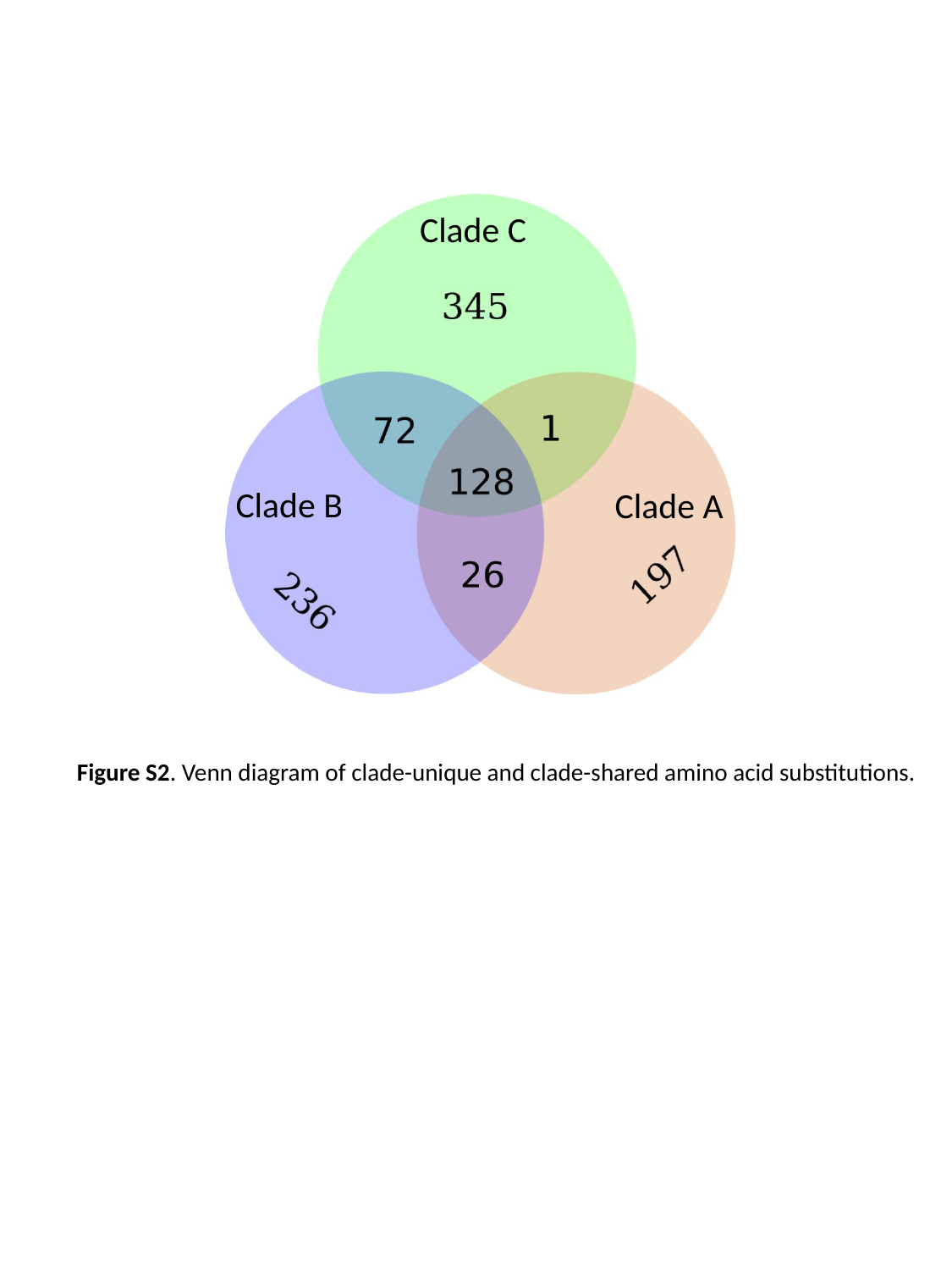

Clade C
Clade B
Clade A
Figure S2. Venn diagram of clade-unique and clade-shared amino acid substitutions.
