# Supplementary material for: New epidemic cluster of pre-extensively drug resistant isolates of Mycobacterium tuberculosis Ural family emerging in Eastern Europe
Source: BMC Genomics. 2018 Oct 22;19:762. doi: 10.1186/s12864-018-5162-3 (PMC6198502; doi:10.1186/s12864-018-5162-3)
Supplement: Supplementary file 4 — Figure S3. WGS-based dendrogram of the Ural strains with added resistance profiles and mutations (see also Additional file 1: Table S2). (PPTX 2084 kb) [file 12864_2018_5162_MOESM4_ESM.pptx]

## Slide 1
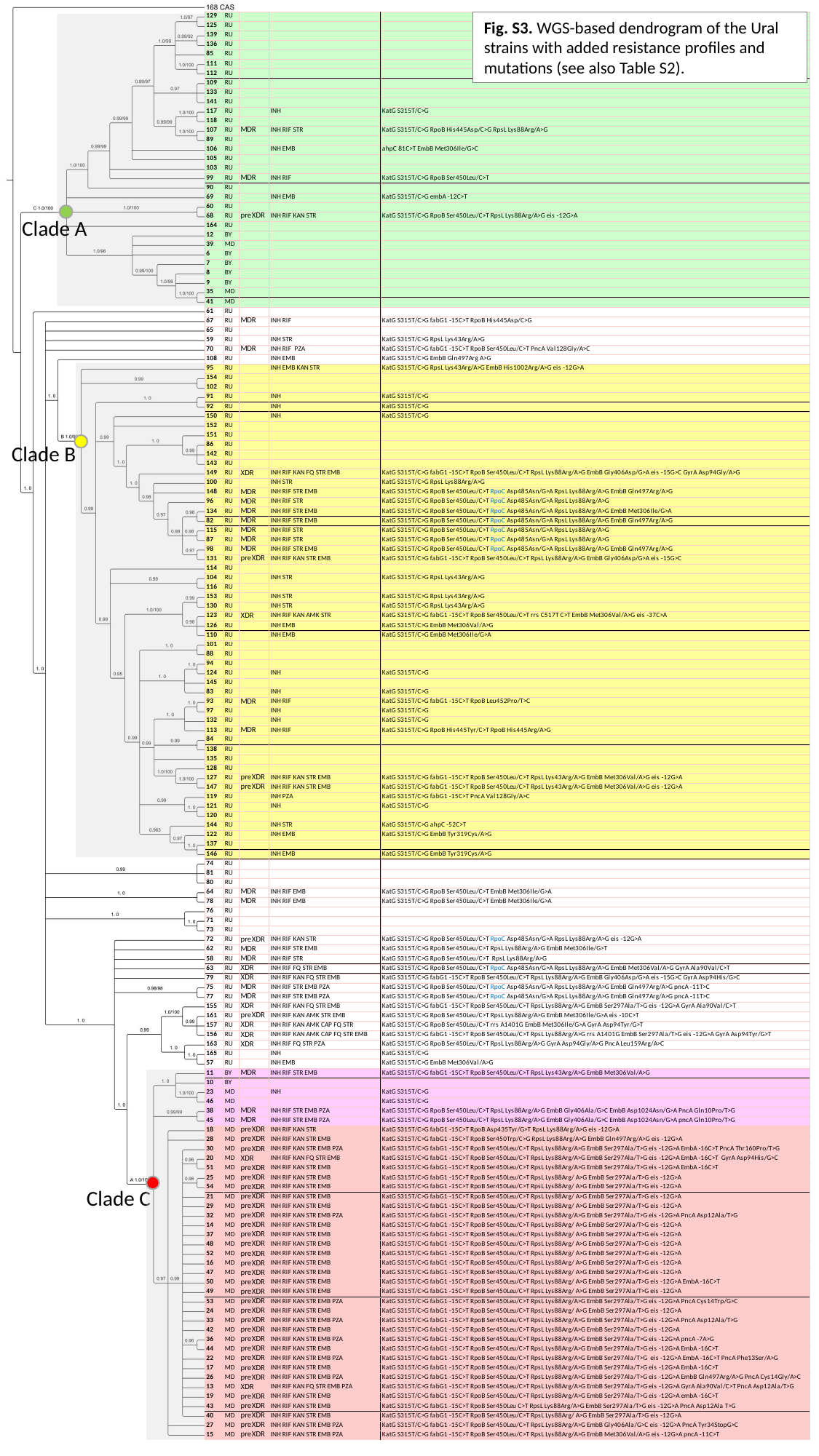

Fig. S3. WGS-based dendrogram of the Ural strains with added resistance profiles and mutations (see also Table S2).
Clade A
Clade B
Clade C
