# Supplementary material for: New epidemic cluster of pre-extensively drug resistant isolates of Mycobacterium tuberculosis Ural family emerging in Eastern Europe
Source: BMC Genomics. 2018 Oct 22;19:762. doi: 10.1186/s12864-018-5162-3 (PMC6198502; doi:10.1186/s12864-018-5162-3)
Supplement: Supplementary file 5 — Figure S4. WGS-based dendrogram of the Ural strains with added spoligotyping profiles. SIT – spoligotype international type according to SITVIT_WEB. Spoligotype signatures specific of the entire Ural family and its specific clades are shown in different colors. (PPTX 2071 kb) [file 12864_2018_5162_MOESM5_ESM.pptx]

## Slide 1
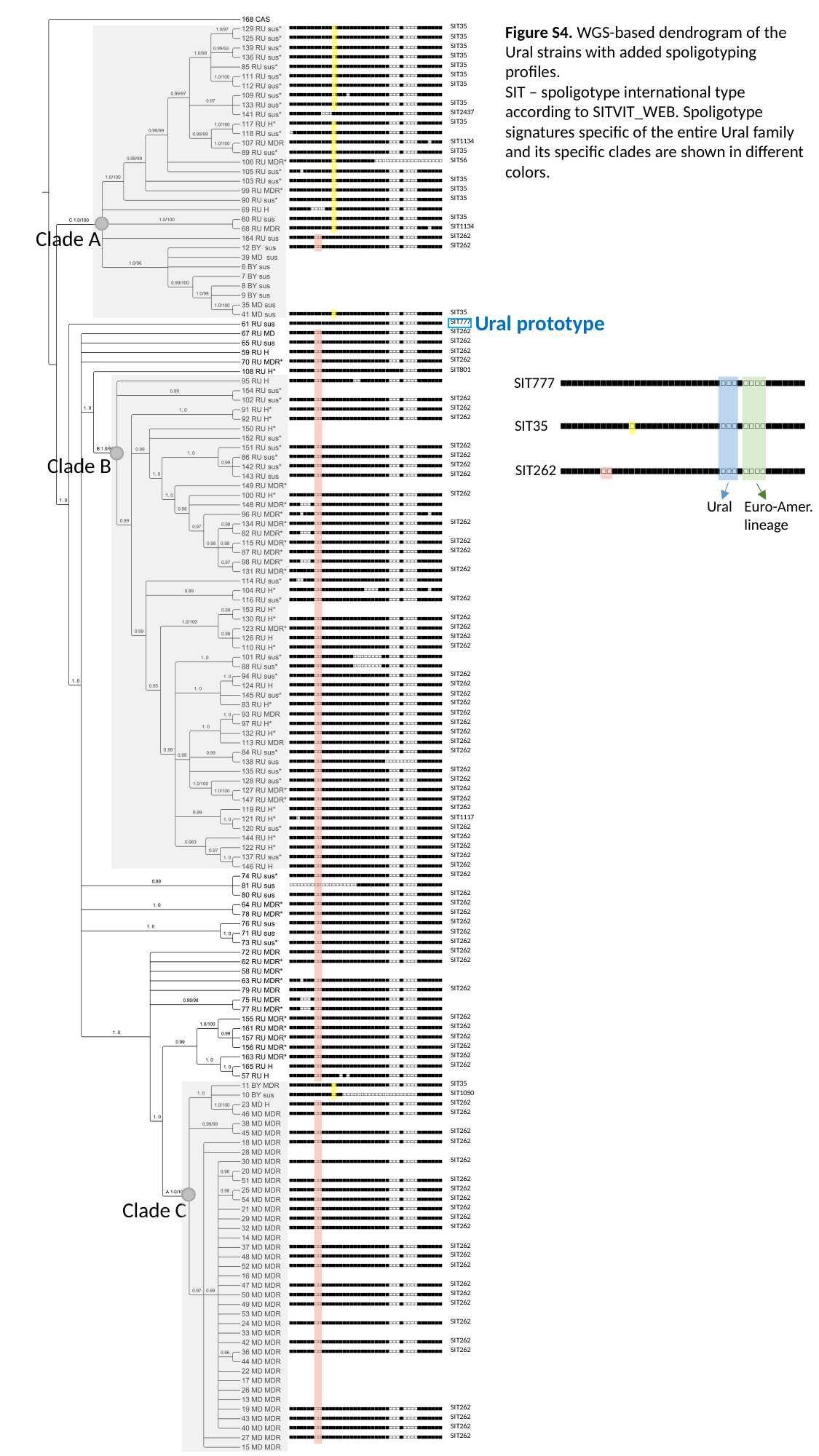

Figure S4. WGS-based dendrogram of the Ural strains with added spoligotyping profiles.
SIT – spoligotype international type according to SITVIT_WEB. Spoligotype signatures specific of the entire Ural family and its specific clades are shown in different colors.
Clade A
Ural prototype
SIT777
SIT35
SIT262
Ural
Euro-Amer.
lineage
Clade B
Clade C
